# Photodynamic Therapy with an Association of Methylene Blue and Toluidine Blue Promoted a Synergic Effect against Oral Squamous Cell Carcinoma

**DOI:** 10.3390/cancers15235509

**Published:** 2023-11-22

**Authors:** Évilin Rocha, Larissa Bomfim, Sérgio Junior, Gustavo Santos, Cássio Meira, Milena Soares

**Affiliations:** 1 Department of Life Sciences, State University of Bahia, 2555, Salvador 41150-000, BA, Brazil; 2 Gonçalo Moniz Institute, Oswaldo Cruz Foundation (FIOCRUZ), 121, Salvador 40296-710, BA, Brazil; 3 Institute of Innovation in Advanced Health Systems (ISI SAS), University Center SENAI/CIMATEC, 1845, Salvador 41650-010, BA, Brazil; 4 Institute of Health Sciences, Federal University of Bahia, Salvador 40231-300, BA, Brazil; gmpires-santos@hotmail.com

**Keywords:** anticancer therapy, phenothiazines, oral carcinoma

## Abstract

**Simple Summary:**

Among the most malignant cancers, oral squamous cell carcinoma stands out as the most common malignant head and neck tumor, with high prevalence around the world, mainly in developing countries. Despite advances in the field of treatment, the prognosis of patients with oral squamous cell carcinoma remains poor, with a 5-year survival rate of less than 50%. Aiming to overcome the limitations of currently existing therapies, the present work proposes to investigate the potential of photodynamic therapy with methylene blue or toluidine blue. We found higher incorporation rates of both drugs in tumor cells compared to the non-tumor cell line. In addition, photodynamic therapy increases cytotoxic activity of both compounds for all lineages evaluated. Moreover, combination of both compounds promotes a synergistic effect on the viability of oral squamous cell carcinoma, opening up possibilities for the use of photodynamic therapy against other cancer cell types using combinations of these photosensitizers.

**Abstract:**

Among the most malignant cancers, oral squamous cell carcinoma (OSCC) stands out as the most common malignant head and neck tumor. Despite advances in the field of treatment, the prognosis of patients with OSCC remains poor. Aiming to overcome the limitations of the currently existing therapies against OSCC, the present work aims to investigate the potential of photodynamic therapy (PDT) with phenothiazine derivatives used alone or in combination. The incorporation of methylene blue (MB) and toluidine blue (TB) was evaluated in OSCC cell lines (HSC-3 and SCC-9) and a nontumor cell line (Hfib). Both compounds exhibited concentration and time-dependent incorporation, with higher rates observed in tumor cells. Regarding dark-phase cytotoxic activity, SCC-9 cells were the most sensitive cell line with an IC_50_ value of 362.6 µM and 41.4 µM for MB and TB, respectively. Using PDT, all lineages showed greater sensitivity, presenting lower IC_50_ values when compared to the dark phase values. The combination index values of 0.69 (dark phase) and 0.73 (clear phase) associated with concave isobolograms, in both phases, revealed that MB and TB have synergistic effects when combined against SCC-9 cells. These findings suggest that MB or TB assisted with PDT holds promise for OSCC treatment.

## 1. Introduction

Among the most malignant cancers, oral squamous cell carcinoma (OSCC) stands out as the most common malignant head and neck tumor, with high prevalence around the world, mainly in developing countries [1,2]. OSCC comprises more than 90% of oral cancers and approximately two thirds of cases occur in developing countries [2]. The first-line treatment is surgical resection associated with chemotherapy or radiotherapy, which can be performed before or after the surgical procedure [3]. Despite advances in the field of treatment, the prognosis of patients with OSCC remains poor, with a 5-year survival rate of less than 50% being reported in the literature [4]. Therefore, new therapies for OSCC are still required.

An attractive option for the treatment of OSCC is the use of photodynamic therapy (PDT). PDT involves the use of a photosensitizer along with a special type of light that, when combined, induce the production of a form of oxygen that is harmful to various cell types, including tumor cells [5,6,7]. PDT presents itself as a potentially applicable, safe and accessible option, being an attractive option compared to the use of conventional chemotherapy, mainly with regard to not inducing resistance and not requiring multiple treatment sessions [8]. PDT is a minimally invasive therapeutic modality that is based on the administration (systemic or topical) of a photosensitizer followed by light activation. The photosensitizer is a type of photoactive pigment that generates singlet oxygen by irradiating visible light using a wavelength that corresponds to its absorption spectrum [9,10]. The activated photosensitizer can react with molecules in its vicinity by transferring electrons or hydrogen, leading to the production of free radicals (type I reaction) or by transferring energy to oxygen, leading to the production of singlet oxygen, causing damage in a more effective way in tumor cells than in normal cells, resulting in the selective death of these cells via apoptotic or necrotic mechanisms (type II reaction). Both pathways can lead to cell death and destruction of diseased tissue [11]. This therapeutic approach has been shown to be a promising alternative for the treatment of several histotypes of cancer, such as: oropharyngeal cancer, esophageal cancer and cutaneous carcinoma [12,13,14].

Among the various classes of photosensitizers, phenothiazines stand out for being molecules with great potential and propensity for the formation of reactive oxygen species [15]. So far, more than 5000 phenothiazine derivatives have been obtained and this class has been highlighted for its variety of chemical and biological properties, low cost and use associated with few adverse effects, since it is activated by ambient light and quickly eliminated from the body [16]. Within the class, methylene blue (MB) and toluidine blue (TB) stand out for their use in the medical and dental fields, both for diagnosis (histopathological stains, tumor identification) and for treatments of diseases such as: cancer, septic shock, malaria, methemoglobinemia and Alzheimer’s [17,18,19,20,21]. Aiming to overcome the limitations of currently existing therapies for OSCC, the present work proposes to investigate the potential of PDT with phenothiazine derivatives used alone or in combination.

## 2. Materials and Methods

### 2.1. Drugs

Methylene blue (MB) and toluidine blue (TB) were purchased from Sigma-Aldrich (St. Louis, MO, USA). Both compounds were diluted in PBS and filtered with a 0.22 µm syringe filter. Both compounds were stored in a −20 °C freezer until use.

### 2.2. Cell Culture

HSC-3 (human tongue squamous cell carcinoma) and SCC-9 (human tongue squamous cell carcinoma) cell lines were obtained from the American Type Culture Collection (ATCC, Manassas, VA, USA). Both cell lines were cultured in DMEM/F12 medium (1:1 mixture of Dulbecco’s modified Eagle’s medium and Ham’s F12 medium, Waltham, MA, USA,) supplemented with 10% fetal bovine serum (FBS; GIBCO-BRL, Gaithersburg, MD, USA), 400 ng/mL hydrocortisone (Sigma-Aldrich) and 50 µg/mL of gentamicin (Life, Carlsbad, CA, USA). The normal human epithelial fibroblast cell line (Hfib) was isolated from a healthy donor skin biopsy and cultured in DMEM medium (GIBCO) supplemented with 10% FBS and 50 µg/mL of gentamicin, as previously described [22]. The cells were maintained in a humidified incubator at 37 °C and 5% CO_2_. All cell lines were regularly tested for mycoplasma contamination using MycoAlert^®^ Mycoplasma Detection Kit (Lonza, Basel, Switzerland).

### 2.3. MB and TB Incorporation Test

Initially, all the cell lines were plated into 96-well plates at a cell density of 2 × 10^4^ cells/well in their respective medium described above for 24 h at 37 °C and 5% CO_2_. After that time, different concentrations of MB (12.5–400 µM) or TB (6.25–200 µM) were added to the plate, in triplicate, and the plates were incubated for different times (15, 30, 60 and 120 min). Then, the plates were washed twice with PBS and the cells were incubated with sodium dodecyl sulfate (SDS; Sigma-Aldrich; 100 µL of a solution of 50 mM diluted in PBS) to promote cell membrane lysis and release of MB or TB incorporated by the cells for 30 min in room temperature. The absorbance of the incorporated MB or TB was measured in a spectrophotometer at a wavelength of 660 nm. Three independent experiments were performed.

### 2.4. Cell Viability

**Figure 1:**
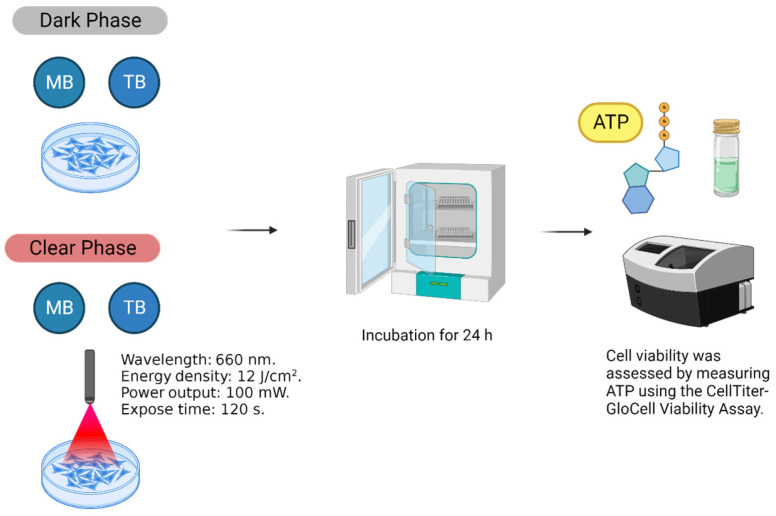
Experiment design. Phenothiazine derivatives (MB and TB) were tested on three cell lines: human fibroblasts (Hfib), HSC-3 (human tongue squamous cell carcinoma) and SCC-9 (human tongue squamous cell carcinoma). Cell viability was measured using CellTiter-Glo^®^ Luminescent Cell viability assay. The experiments were performed in the absence (dark phase) or presence (clear phase) of PDT.

The cells were plated into 96-well plates at a cell density of 2 × 10^4^ cells/well in in their respective medium described above for 24 h at 37 °C and 5% CO_2_. After that time, different concentrations of MB (12.5–400 µM) or TB (6.25–200 µM) were added to the plate, in quadruplicate, and the plates were incubated were at 37 °C for 2 h. Then, the plates were washed twice with PBS, the medium was replaced, and the plates were incubated at 37 °C for an additional 24 h. Finally, cell viability was measured using CellTiter-Glo^®^ Luminescent Cell viability assay, which indicates cell viability by measuring ATP, following the manufacturer’s instructions. Both compounds were tested in the absence of light (dark phase) or after photodynamic exposure (clear phase) with a diode laser which has a semiconductor active medium InGaAlP (λ 660 nm, 100 mW, 12 J/cm^2^, CW, Flash Lase III, DMC equipamentos, São Carlos, São Paulo, Brazil) (Figure 1). The photodynamic exposure was carried out after the washes with PBS in wells with 200 µL of DMEM.

### 2.5. Drug Combination Assay

For in vitro drug combinations, each one of the drugs was tested in eight concentrations alone (12.5–400 µM) or combined in a ratio of 1:1 or 2:1 MB/TB (3.12–200 µM) against SCC-9 cells following the protocol described above. The combined therapy was performed during both the dark and clear phase. The analysis of the combined effects was performed by determining the combination index (CI), used as cutoff to determine synergism, by using the Chou–Talalay CI method [23], and through the construction of an isobologram using the fixed ratio method, as described previously [24].

### 2.6. Statistical Analyses

We used non-linear regression for calculating the IC_50_ values. One-way analysis of variance and Newman–Keuls multiple comparison tests were used to determine the statistical significance of the group comparisons in the in vitro assays. The results were considered statistically significant when *p* was <0.05. All analyses were performed using Graph Pad Prism version 8.0 (Graph Pad Software, San Diego, CA, USA).

## 3. Results and Discussion

**Figure 2:**
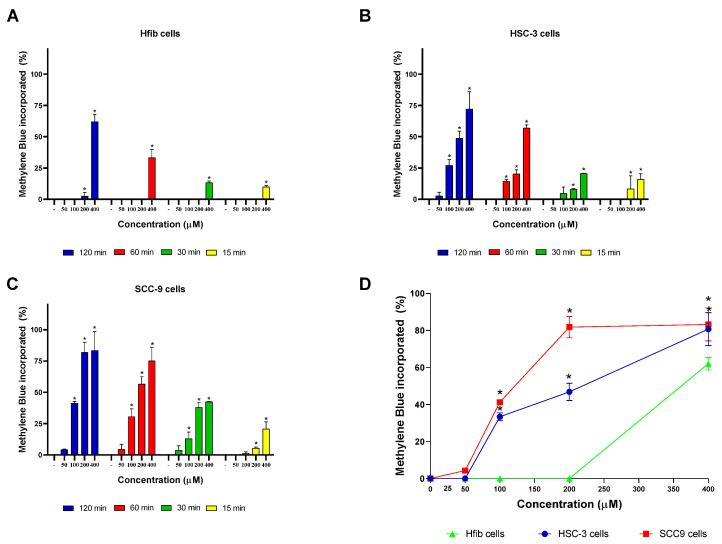
MB incorporation over time in different cell lines. (**A**) Incorporation rate of MB in Hfib cells. (**B**) Incorporation of MB in HSC-3 cells. (**C**) Incorporation of MB in SCC-9 cells. (**D**) Comparison of the MB incorporation rate between the cell lines evaluated after 2 h of treatment. Values represent the means ± SD of four determinations obtained in one of the three experiments performed. * *p* < 0.05 compared to untreated cells (-) in graphs (**A**–**C**). * *p* < 0.05 compared to the incorporation rate of MB by Hfib in the same concentration in graph (**D**).

Initially, the incorporation of phenothiazine derivatives (MB and TB) was determined in oral squamous cell carcinomas (HSC-3 and SCC-9) and in a non-tumor cell line (Hfib). As revealed in Figure 2, MB incorporation showed a concentration and time-dependent profile for all cell lines, with the highest incorporation rates after two hours of incubation for all lineages. Interestingly, significant rates (*p* < 0.05) of MB incorporation by non-tumor cells were only observed at the highest concentration evaluated (400 µM), which is different to what was observed in tumor cells that had significant rates (*p* < 0.05) of MB incorporation at concentrations ranging from 100 to 400 µM (Figure 2). Among the three evaluated lineages, the SCC-9 cell line showed the highest MB incorporation rates (Figure 2).

**Figure 3:**
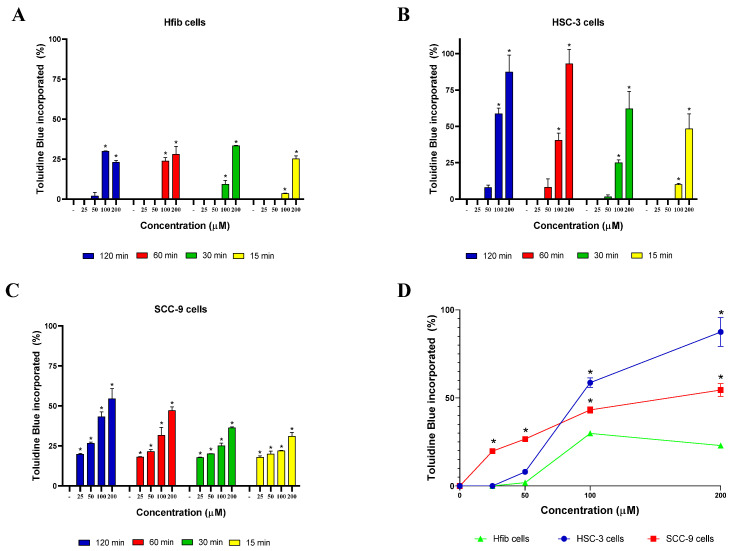
TB incorporation over time in different cell lines. (**A**) Incorporation of TB in Hfib cells. (**B**) Incorporation of TB in HSC-3 cells. (**C**) Incorporation of TB in SCC-9 cells. (**D**) Comparison of the TB incorporation rate between the cell lines evaluated after 2 h of treatment. Values represent the means ± SD of four determinations obtained in one of the three experiments performed. * *p* < 0.05 compared to untreated cells (-) in graphs (**A**–**C**). * *p* < 0.05 compared to the incorporation rate of TB by Hfib in the same concentration in graph (**D**).

The TB incorporation rate was also evaluated in the three cell lines. An incorporation profile similar to that of MB was observed, with the two tumor cell lines having more significant incorporation rates (*p* < 0.05) than the non-tumor cell line, especially at 100 or 200 µM (Figure 3). A concentration and time-dependent incorporation of TB was also observed in the tumor cell lines, with the time of 2 h showing the highest TB incorporation rates for both tumor cell lines. Regarding the lineage with the highest TB incorporation rates, we observed that at high concentrations (100 and 200 µM), the HSC-3 lineage had greater absorption and at lower concentrations (25 and 50 µM) the SCC-9 cell line had higher rates of incorporation (Figure 3).

The tendency of both compounds being better incorporated by tumor cells than non-tumor cells is well described in the literature [25,26,27]. This feature justifies the employment of both compounds as a visual tumor marker to detect and delineate certain types of cancers, especially on oral cavity, and their use as photosensitizers for the treatment of different types of cancer [28,29]. The selectivity of the evaluated phenothiazine derivatives may be related to the compounds’ affinity for low-density lipoproteins (LDLs), which are found in greater amounts in tumor cells [30]. LDLs promote the cholesterol uptake that is necessary for membrane formation during cell division and work as a “transporter” of the photosensitizers into intracellular space [31,32]. Once tumor cells have increased mitotic division rates, they have a high expression of lipoprotein receptors on the cell surface and a higher LDL uptake [30,33].

**Figure 4:**
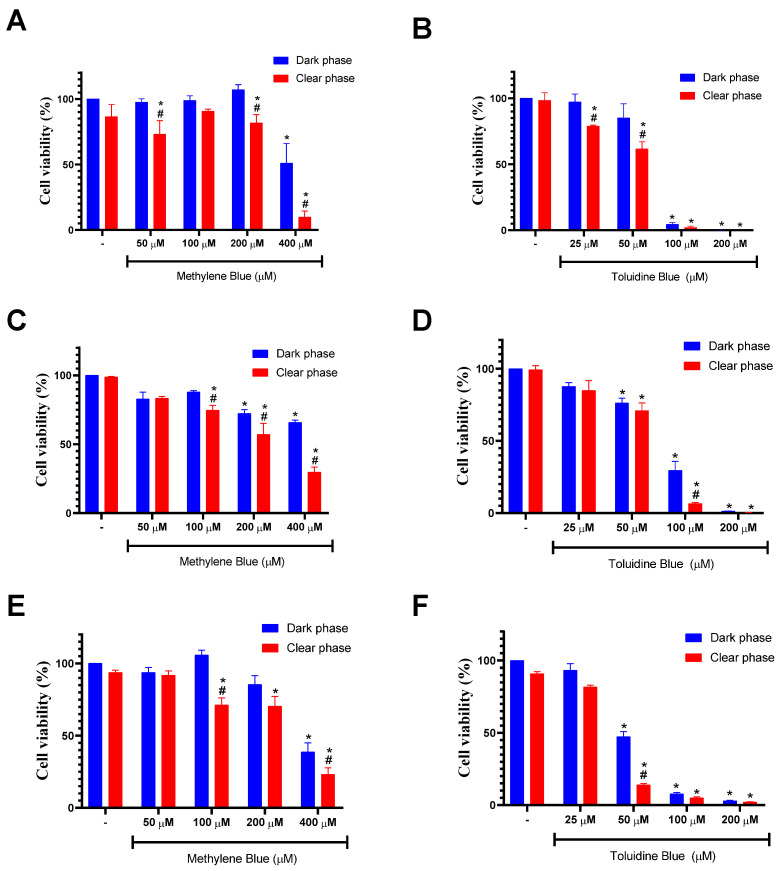
Effects of MB and TB on the cell viability of Hfib (**A**,**B**), HSC-3 (**C**,**D**) and SCC-9 cell lines (**E**,**F**). Cells were treated with different concentrations of methylene blue (50–400 µM) or toluidine blue (25–200 µM) in the absence (dark phase; blue columns) or presence (clear phase; red columns) of PDT. Cell viability was determined by ATP measurement using the CellTiter-Glo Luminescent Cell Viability Assay. Values represent the means ± SD of four determinations obtained in one of the three experiments performed. * *p* < 0.05 compared to untreated cells (-). # *p* < 0.05 compared to the same concentration in the dark phase.

Regarding cytotoxic activity in the dark phase, MB showed a significant inhibitory effect on cell viability, especially at 400 µM for all lineages, with the SCC-9 cell line being the most sensitive with an IC_50_ value of 362.6 µM (Figure 4; Table 1). After using a fixed energy density (J/cm^2^) to excite MB, we found an increase in cytotoxic effect, with IC_50_ values of 234.5, 307.4 and 294.4 µM against HSC-3, SCC-9 and Hfib cells, respectively, versus IC_50_ values of >400, 362.6 and >400 in the dark phase, respectively (Table 1). The largest difference was observed in HSC-3 cells at 400 μM. When tested in the absence of light, MB (400 μM) caused an inhibition of 34.3% in cell viability. Under the same conditions, however, in the presence of light, MB (400 μM) inhibited 70.3% of cell viability (Figure 4). The difference observed between the groups was statistically significant (*p* < 0.05). MB-PDT is well employed against several types of cancer such as: cervical carcinoma, leukemia, osteosarcoma, mammary adenocarcinoma, melanoma and lung adenocarcinoma [7,29,34,35,36]. However, MB-PDT effects on OSCCs are poorly described and also evaluated with methodologies less sensitive than the ATP-based assay as was used in the present work [37,38,39]. Previously, KOFLER and colleagues [37] demonstrated cytotoxic activity of MB alone or with PDT in CA-9-22 cells, and LE and colleagues [38] showed the cytotoxic activity of MB in SCC-25 cells.

In comparison to MB, TB showed a more prominent cytotoxic effect on cell viability in the dark phase for all lineages, especially in concentrations between 50 and 200 µM (Figure 4). As revealed in Table 1, the lowest IC50 value found was obtained using the SCC-9 cell line (41.4 µM). The dark toxicity of TB is well characterized in the literature and also associated with undesired side effects, thereby limiting the concentrations and doses that can be used [40,41,42].

In the clear phase, all lineages showed greater sensitivity, presenting lower IC_50_ values when compared to the dark phase values, again with the SCC-9 cell line showing the highest sensitivity with an IC_50_ value of 33.8 µM (Table 1). The largest difference was observed in SCC-9 cells at 50 μM. When tested in the dark phase, TB caused an inhibition of 52.6% in cell viability. Under the same conditions, however, in the presence of light, TB inhibited 86% of cell viability (Figure 4). The difference observed between groups was statistically significant (*p* < 0.05). Similar results using TB-PDT were found using breast cancer cell lines, where TB presented an IC_50_ value of 1.13 µM in the dark phase and 0.88 µM in the presence of light, reinforcing the advantages of PDT [41]. Despite its already established use in conjunction with other techniques in the field of cancer diagnosis, the use of TB as a photosensitizer for the treatment of oral cancer is not much discussed in the literature [42]. It is also important to report that exposure to PDT without a photosensitizer did not cause loss of cell viability in the three lineages evaluated, suggesting that the phototoxicity must be attributed exclusively to phenothiazine derivatives as previously reported [41,43].

Lastly, the cytotoxic activity of MB and TB in combination was investigated against SCC-9 cells, the most sensitive lineage for both compounds. First the combination was carried out in the absence of light, where MB and TB presented IC_50_ values of 368.5 and 43.8 µM when tested individually, and 30.5 and 26.9 µM when tested in combination, respectively. These data demonstrate an approximately 13-fold reduction in the IC_50_ value for MB and an approximately 2-fold reduction in the IC_50_ value for TB (Table 2).

Drug combination was also performed in the presence of light. As shown in Table 2, in the clear phase, MB and TB showed IC_50_ values of 302.3 and 33.7 µM when tested separately and 23.4 and 22.4 µM when combined, respectively. These data demonstrate an approximately 13-fold reduction in the IC_50_ value for MB and an approximately 1.5-fold in the IC_50_ value for TB.

**Figure 5:**
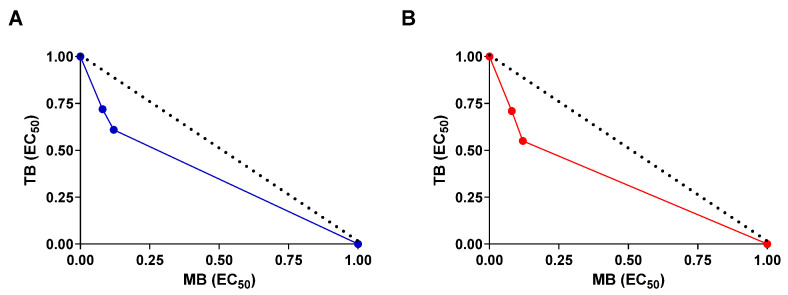
Isobologram describing the synergistic effects of MB and TB on SCC-9 cell viability. The test was conducted in the absence of light (dark phase) (**A**) or in the presence (clear phase) of PDT (**B**). Cell viability was determined by ATP measurement after 24 h of incubation. Broken lines correspond to the predicted positions of the experimental points for additive effects.

Moreover, the combination index values of 0.69 (dark phase) and 0.73 (clear phase) associated with concave isobolograms, in both phases, revealed that MB and TB have synergistic effects when combined against the cell viability of SCC-9 cells (Table 2; Figure 5). Interestingly, the synergistic effect of MB and TB in the dark phase has already been demonstrated on the inhibition of the proliferation of amastigotes of *T. cruzi* with a combination index of 0.89 [44]. In addition, combination of MB plus TB in the clear phase eliminated the bacterial growth of *Xanthomonas campestris* pv. campestris found in naturally contaminated canola seeds, which did not occur when the photosensitizers were tested alone [45]. For treatment of OSCC, this is the first report related to the synergic effect of a combination of MB plus TB in the dark phase and clear phase. However, combinations of phenothiazine derivatives assisted with photodynamic therapy already show synergistic effects with other molecules and with recognized cytotoxic activity. For example, combination therapy with MB-assisted photodynamic therapy and salicylic acid or with doxorubicin against breast cancer cells improve the efficacy of chemotherapy [46,47].

## 4. Conclusions

In sum, MB or TB assisted with PDT as a useful strategy for the treatment of oral carcinoma was reinforced in the present investigation. In addition, it was shown that the combination of MB and TB has a synergistic effect on the viability of oral squamous cell carcinoma, opening up possibilities for the use of PDT against other cancer cell types using combinations of these phenothiazine derivatives already well characterized as promising photosensitizers.

## Figures and Tables

**Table 1:** Cytotoxic activity of MB and TB in different cell lines in the absence (dark phase) or presence (clear phase) of light.

Sample	IC_50_ ± S.D (μM) HSC-3		IC_50_ ± S.D. (μM) SCC-9		IC_50_ ± S.D. (μM) Hfib	
	Dark Phase	Clear Phase	Dark Phase	Clear Phase	Dark Phase	Clear Phase
MB	>400	234.5 ± 9.6	362.6 ± 15.6	307.4 ± 28.8	>400	294.4 ± 12.1
TB	73.2 ± 2.6	58.7 ± 2.3	41.4 ± 4.0	33.8. ± 1.4	66.6 ± 5.9	53.7 ± 3.6

Values represent the mean ± SD and were calculated from three independent experiments. IC_50_ = inhibitory concentration of 50%. MB = methylene blue. TB = toluidine blue. SD = standard deviation.

**Table 2:** Cytotoxic activity of MB and TB on SCC-9 cells alone or in combination.

Sample	IC_50_ ± S.D. (μM) Dark Phase		IC_50_ ± S.D. (μM) Clear Phase		CI ^a^	
	Drug Alone	Combination	Drug Alone	Combination	CI Dark Phase	CI Clear Phase
MB	368.5 ± 10.8	30.5 ± 4.8	302.3 ± 25.7	23.4 ± 1.4	0.69 ± 0.05	0.73 ± 0.08
TB	43.8 ± 2.9	26.9 ± 1.4	33.7 ± 1.2	22.4 ± 2.4		

Values represent the mean ± SD and were calculated from two independent experiments. ^a^ Combination index (CI). Cutoff: CI value of 0.3–0.7, synergism; 0.7–0.85, moderate synergism; 0.85–0.9, slight synergism; 0.9–1.1, additivity; > 1.1, antagonism. IC_50_ = inhibitory concentration of 50%. MB = methylene blue. TB = toluidine blue. SD = standard deviation.

## Data Availability

The data presented in this study are available on request from the corresponding author.
